# A comparison between veterinary small animal general practitioners and emergency practitioners in Australia. Part 1: demographic and work-related factors

**DOI:** 10.3389/fvets.2024.1355505

**Published:** 2024-03-21

**Authors:** Kun Li, Erin Mooney, Michelle McArthur, Evelyn Hall, Anne Quain

**Affiliations:** ^1^Sydney School of Veterinary Science, Faculty of Science, The University of Sydney, Camperdown, NSW, Australia; ^2^School of Animal and Veterinary Science, Faculty of Sciences, Engineering and Technology, University of Adelaide, Adelaide, SA, Australia

**Keywords:** burnout, veterinary, mental health, workplace risk-factors, Copenhagen Burnout Inventory

## Abstract

Occupational stressors are commonly encountered in small animal veterinary practice and have been associated with burnout. The working context of veterinarians differs by specialty, and this can potentially lead to variable exposures to risk factors for burnout. The aim of this study was to explore differences in demographic and working conditions of veterinary general practitioners (GPs) and emergency practitioners (EPs) to compare exposure to different potential stressors. An anonymous, online survey was administered to veterinary GPs and EPs practicing in metropolitan regions of Australia. In total, 320 participant responses were analyzed (*n* = 237, 74.2% GPs and *n* = 83, 25.9% EPs). Significant differences (*P* < 0.05) in the demographics and work-related exposures were found between the two groups. GPs were found to be older than EPs with a greater number of years of experience in their field (*P* < 0.001). Most veterinary GPs worked only day shifts (207/236, 87.7%); where EPs worked a greater variety of shift patterns, with “only day shifts” being the least common shift pattern (*P* < 0.001). Most GPs worked a set and predictable roster pattern (195/236, 83.6%), while most EPs did not (51/83, 61.5%). EPs worked more weekends and public holidays (*P* < 0.001). The EP group performed more hours of work each week but worked less overtime. The main contributing factors for overtime were scheduling factors for GPs and staffing issues for EPs. EPs were commonly not able to take meal-breaks and GPs' meal-breaks were commonly interrupted by work. EPs were more frequently exposed to patient death, euthanasia (including for financial reasons), emotionally distressed clients and delivering negative news (*P* < 0.001). Both groups indicated that most work environments were collegiate and supportive, and a minority reported toxic colleagues (11.8%) or management teams (26.9%). Just under one-half of respondents reported having witnessed or experienced workplace bullying. Of our respondent group, 52.0% (166/319) were not satisfied with their remuneration. Desire to leave their principal area of practice was prevalent among this survey group (192/319, 60.2%) with approximately one-third considering leaving the veterinary profession. We discuss the implications of these workplace factors, including mitigation strategies.

## Introduction

Veterinarians are exposed to many stressors through their daily work (1–5). With time, these stressors compound and can lead to burnout (6, 7). Burnout is an occupational syndrome that is characterized by three dimensions: overwhelming exhaustion; increased cynicism and reduced efficacy toward work. Burnout, stress and suicidal ideation have been documented in the veterinary sector (8–11). The prevalence of burnout and stress among veterinarians range from 23% to 66.3%, depending on jurisdiction (12–18). Burnout in veterinarians is associated with absenteeism, attrition, lower level of patient care and increased errors (19–21). A 2022 study estimated $1–2 billion of annual revenue losses could be attributed to burnout of veterinarians in the United States (22). This is likely an underestimation of the financial cost of burnout as the mathematical model could only account for losses due to turnover and reduced working hours. On an individual level, burnout is associated with increased risk of injury, insomnia, substance abuse, and depression (23).

Despite studies showing the high prevalence of burnout among veterinarians (12–16), there is limited research investigating the relationship between the specific type of veterinary practice and burnout. A 2016–2018 US survey study found that veterinarians who spent <25% of their time working with small animals (cats and dogs) had a significantly lower mean burnout score when compared to veterinarians who worked 75% of the time or more with small animals (24). In contrast, a Belgian study comparing practitioners in mixed, small animal and bovine-only practice found a similar level of burnout across all groups (15). This was echoed in an Australian study comparing small animal, mixed-practice, large animal and non-clinical veterinarians, which did not find any relationship between mental health and type of practice (25). However, as veterinary medicine advances, and pet owners' expectations grow, small animal practice becomes increasingly sub-specialized (26–28). The caseload and working context of veterinarians differ between practice and specialty types, potentially leading to variability in exposure to risk factors for burnout. As burnout is an occupational syndrome, it is useful to understand potential workplace risk factors that may be associated with burnout, and how exposures to these factors differ between different groups.

Emergency physicians experience higher levels of burnout compared to other human physicians (29, 30), while general practitioners had lower rates of burnout (31). Burnout was correlated with shift work, sleep disturbances, longer working hours, weekend shift work, work-family conflict and quality of teamwork (30, 32, 33), which may be more prevalent for veterinary emergency practitioners (EPs) than general practitioners (GPs). However, there is scant published information comparing workplace factors between GPs and EPs. The first part of this two-part study explores differences between demographic and working conditions of GPs and EPs.

## Materials and methods

### Survey

A cross-sectional survey design was employed for this study. The survey was split into three sections (see Supplementary Table 1). Questions were designed based on recent literature reporting on risk-factors for burnout among veterinarians (8, 12, 15, 16, 34) and physicians (29, 30, 33), then refined by the research team based on our combined experience as GPs and EPs, and reference to the Australian Animal Care and Veterinary Services Award 2020 (35).

The first section contained a series of 29 questions (25 primary questions and four conditional questions) focused on working conditions pertaining to GPs and EPs. These questions covered the nature of employment, potential work-related risk factors for burnout, job satisfaction and intentions to resign. This section comprised of six binary questions, 20 multiple choice questions and three open-ended questions – “How long have you worked in your principal area of practice (GP/emergency)?”; “In relation to the previous question, on average how many weeks in advance do you receive your roster?” and “If other, please specify” in relation to the previous multiple-choice question of “what is the main contributing factor to not finishing on time?”

The Australian Animal Care and Veterinary Services Award 2020 was consulted to ensure accuracy and relevance of the options provided for the question regarding meal break (“In the past week, the following statement best describes my meal breaks”). Section 14.1 stipulates that “An unpaid meal break of not <30 min must be allowed to each employee between the fourth and fifth hour of work. In times of emergency or staff accident or illness the time of the meal break can be varied by agreement between the employer and the employee.” Therefore, in this study “unable to take a meal break” was defined as having a meal break for fewer than 30 min.

In the second section, participants were presented with the Copenhagen Burnout Inventory (CBI), a widely used psychological instrument (36). Response to every item of the CBI was mandatory for participants who wished to submit the survey. Details regarding the CBI and the results of this section are reported in part two.

The third section of the survey consisted of three demographic questions collecting information regarding the respondents' age, gender, and family make-up (family composition).

The survey was piloted by ten individual veterinarians, recruited through the primary author's professional network. All feedback that improved the clarity of questions was incorporated. The survey was built and administered on Research Electronic Data Capture (REDCap) hosted on The University of Sydney's secure and restricted-access server.

A power calculation was undertaken prior to recruitment which indicated that a sample of 63 respondents in each study group (EP and GP) was required to detect a difference of five points on the CBI (36), assuming standard deviation of 10 units with 80% power with *P* < 0.05.

### Recruitment, consent, and ethics approval

A three-pronged strategy was employed to maximize the recruitment across Australia (37). Firstly, a survey link was shared in the newsletters, websites and social media forums of veterinary peak bodies, state/territory veterinary boards, professional organizations and special interest groups (listed in Supplementary Table 2). Secondly, survey invitations were distributed through the research team's professional networks via email. Finally, respondents were encouraged to share the survey link with their own professional networks; a snowball sampling technique known as an efficient and valid approach for recruiting unknown populations online (37). Participation was voluntary, with no incentives offered. The survey link was available for completion from the 22nd February to 22nd June 2022.

To be eligible for inclusion, respondents were required to be over the age of 18 and a small animal veterinarian practicing in a metropolitan area of Australia as a veterinary EP or GP. In Australia, a metropolitan area is defined as an urban center with a population of 100,000 or more by the Rural, Remote and Metropolitan Areas Classification (RRMA) 1991. This designation was selected to limit other confounding social variables not examined in this survey.

The participant information statement was available to participants as the survey landing page and was also available for download. Consent was indicated by submitting the survey through REDCap. Survey responses were anonymous, and no identifying data were collected as part of this study. Participants wishing to receive a summary of the results were instructed to email the primary author independently. These addresses were stored independently of responses to the main survey. This study was approved by the University of Sydney's Human Research Ethics Committee (HREC), project number 2022/014.

### Data cleaning

Survey data were downloaded from REDCap into Microsoft Excel^®^ Version 2301 (Build 16026.20146) to allow data cleaning. Respondents that had selected “other” and stated a response that was already included in the options were recategorized into the appropriate category. Respondents who had selected “other” and whose responses were not reflected in the drop-down menu were retained. Those that were retained as “other” were grouped into categories based on similarity of responses.

### Statistical analyses

IBM SPSS Statistics version 28 was utilized to collate and calculate the descriptive statistics for all questions, including the frequency of each variable, total number of missing data for each question and the valid percentage for each variable.

For the questions regarding frequency of interaction with emotionally distressed clients; frequency of delivering negative news and frequency of interacting with compliant clients, the categories “rare” and “never” were combined into “rare/never” for the purpose of statistical analysis due to the small number of respondents choosing these categories.

Statistical analyses were conducted in Genstat v 22, VSN International (2022), and a *p* < 0.05 was considered significant. Chi -squared tests for association were used to assess associations between type of veterinarian (GP/EP) and demographic and work-related factors.

## Results

A total of 506 potential respondents viewed the survey between 22nd February and 22nd June 2022. Of these, 122 partially responded but did not indicate consent by pressing the “submit” button and 63 did not answer any survey questions. One participant failed to select a principal area of practice; hence their data could not be utilized in addressing the main study question, therefore their response was also removed from analysis. In total, 320 participants completed the survey, with 237 (74.1%) respondents enrolled as general practitioners and 83 (25.9%) respondents as emergency practitioners. Thus, there were 320 valid responses available for analysis. Of the 320 respondents, 309 answered all questions of the survey.

### Demographics

Out of the three demographic parameters explored, only age was significantly different between GP and EP groups (*P* < 0.001). The mean age of the GP group was 43 years, compared to 37 years in the EP group. There was a lower proportion of GPs in the 30–39 year-old group than EPs (30.1% vs. 55.4%). Conversely, the proportion of GPs in the >50 year-old group more than doubled EPs (29.2% vs. 10.8%).

Gender and family composition were not significantly different between the two groups. Due to the small sample size for respondents identifying as “other” (*n* = 1), this gender group could not be included in subsequent analysis. The majority of respondents were female (80.6%) and were married or in de-factor relationships with (31.9%) or without (39.4%) dependent children. Descriptive statistics regarding demographics can be found in Table 1.

**Table 1:** Frequency table for the demographic information on respondents to mixed methods survey on burnout in GPs and EPs in Australia (*n* = 320).

**Demographic parameter**	***P-*value**	**Category**	**GP number**	**GP %**	**EP number**	**EP %**	**Total number**	**Total %**
Age (*n =* 319)	**< 0.001**	Less than 30 years old	33	14.0	13	15.7	46	14.4
		30 – 39 years old	71	30.1	46	55.4	117	367
		40 – 49 years old	63	26.7	15	18.1	78	24.5
		50 years old and older	69	29.2	9	10.8	78	24.5
Gender (*n =* 319)	0.533	Female	188	79.7	69	83.1	257	80.6
		Male	47	19.9	14	16.9	61	19.1
		Other	1	0.4	0	0	1	0.3
Household composition (*n =* 320)	0.106	Single, no dependent children	55	23.2	25	30.1	80	25.0
		Single, with dependent children	12	5.1	0	0	12	3.8
		Married/de-facto relationship, no dependent children	87	36.7	39	47.0	126	39.4
		Married/de-facto relationship, with dependent children	83	35.0	19	22.9	102	31.9
Years in principal area of practice (*n =* 317)	**< 0.001**	< 5 years	50	21.3	37	45.1	87	27.4
		5–9 years	41	17.5	21	25.6	62	19.6
		10–19 years	64	27.2	15	18.3	79	24.9
		20 years or more	80	34.0	9	11.0	89	28.1
Position in practice (*n =* 320)	0.064	Associate veterinarians	175	73.8	69	83.1	244	76.3
		Management	19	8.0	8	9.6	27	8.4
		Practice owner	43	18.1	6	7.2	49	15.3

Bold values indicate statistically significant, *P*-value < 0.05.

Most respondents indicated that their principal area of practice was general practice (GP) (*n* = 237, 74.1%), while 25.9% (*n* = 83) were in emergency practice (EP). Most respondents were associate veterinarians (76.3 %) and there was no significant difference (*P*=0.064) in “position in practice” between GP and EP groups.

An even spread in experience was captured in this survey, with similar numbers of respondents in each of the categories: <5 years, 5–9 years, 10–19 years and 20 years or more experience (see Table 1). However, there was a significant difference in years of experience between the two groups (*P* < 0.001). Proportionally, 21.3% of GPs had <5 years of experience in their principal area of practice compared to 45.1% of EPs. Proportionally more GPs had >20 years of experience compared to EPs (34.0% vs. 11.0%) (see Table 1).

### Hours of work and shift pattern

Significant differences were found between GP and EP groups in the number of work hours performed per week (*P* = 0.012); day/night shift pattern (*P* < 0.001); frequency of weekend work (*P* < 0.001) and public holiday work (*P* < 0.001).

**Figure 1:**
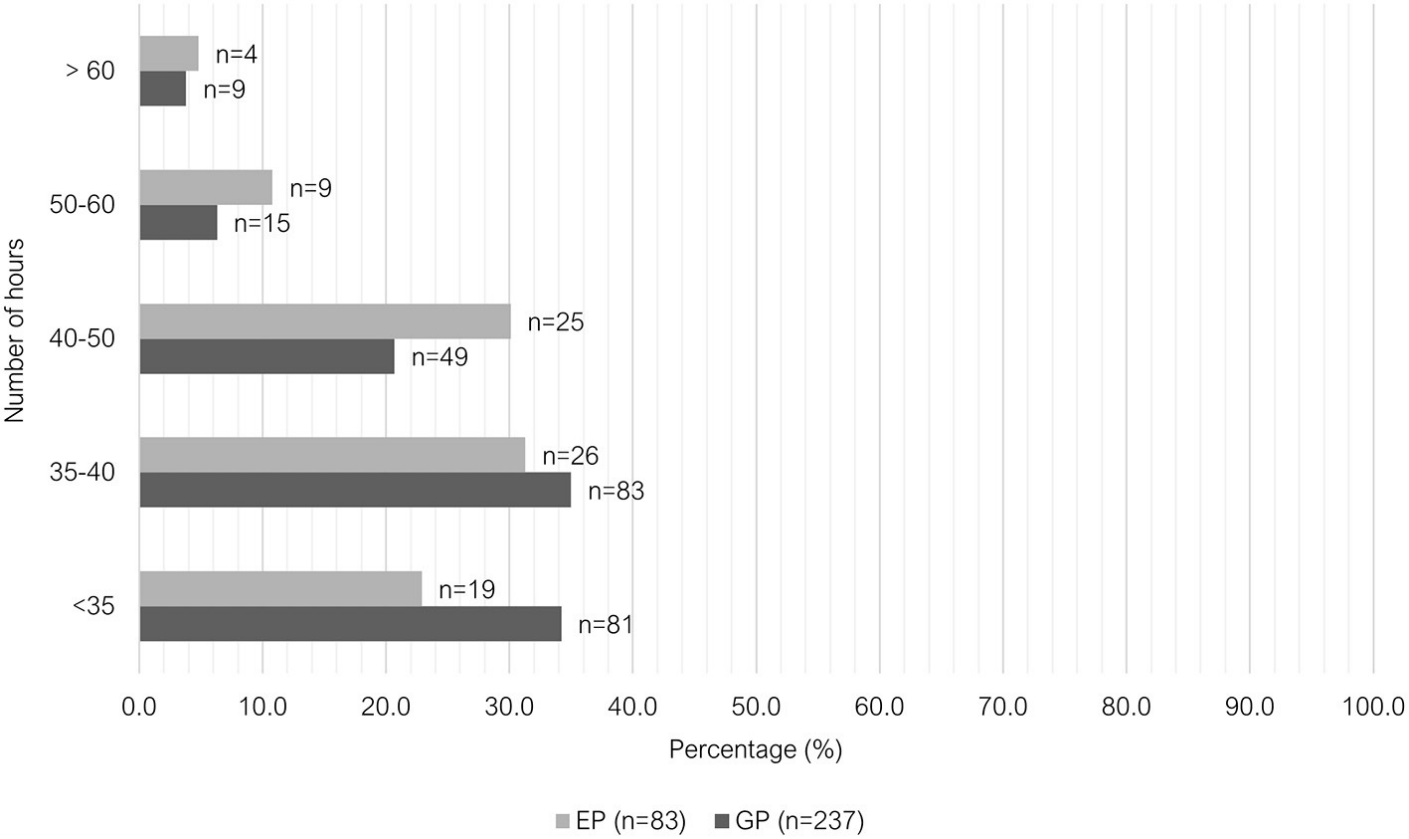
Clustered bar chart for the number of hours worked per week by veterinary GPs and EPs, based on the responses of 320 veterinarians surveyed between February and June 2022.

Most EPs worked between 35 to 40 h per week (26/83, 31.3%), followed closely by 40 to 50 h per week (25/83, 30.1%), and <35 h per week (19/83, 22.9%). Similarly, the largest proportion of GPs worked between 35 to 40 h per week (83/237, 35.0%). However, 34.2% (81/237) of GP vets worked <35 h per week and 20.7% (49/237) worked between 40 to 50 h per week. Relatively, small numbers of vets in both groups worked 50 to 60 h per week or >60 h per week (Figure 1).

**Figure 2:**
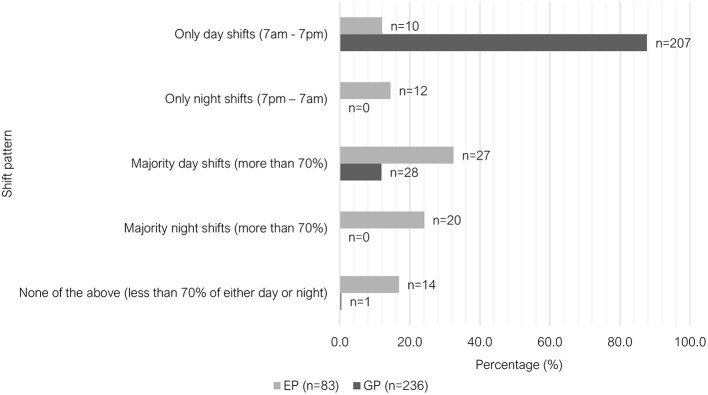
Clustered bar chart for the different shift patterns worked by veterinary GPs and EPs, based on the responses of 320 veterinarians surveyed between February and June 2022.

**Figure 3:**
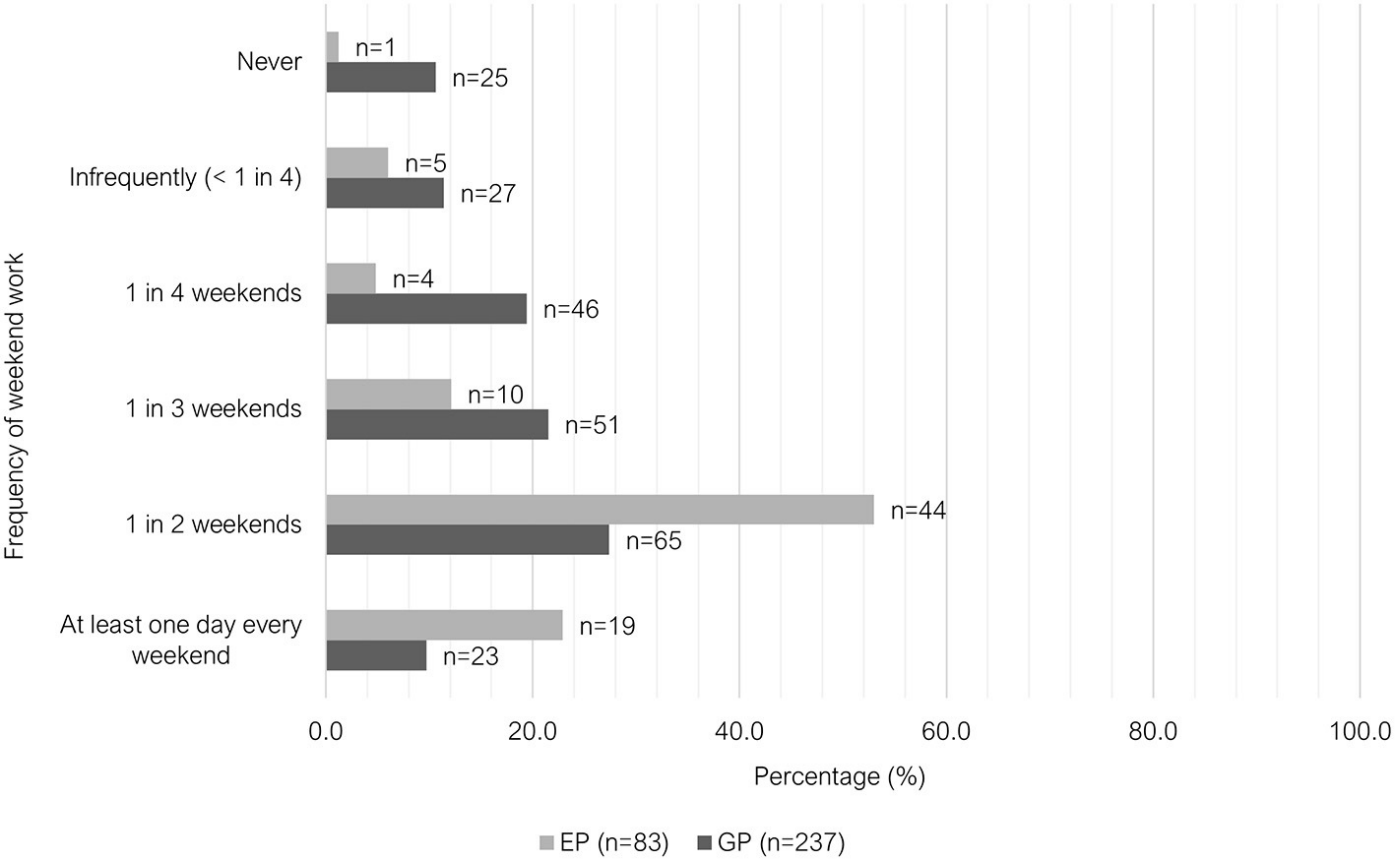
Clustered bar chart for the weekend work patterns of veterinary GPs and EPs, based on the responses of 320 veterinarians surveyed between February and June 2022.

**Figure 4:**
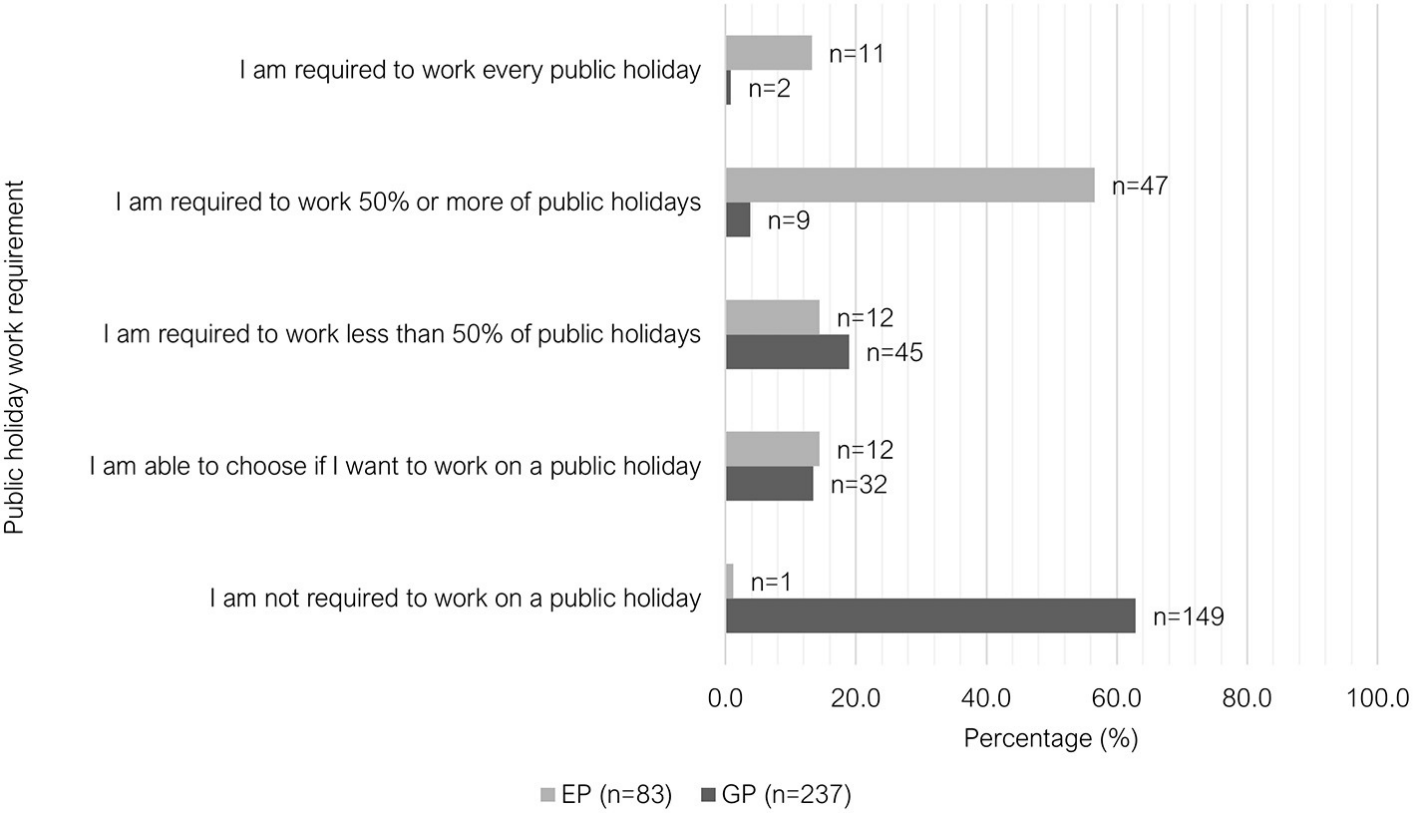
Clustered bar chart for the public holiday work patterns of veterinary GPs and EPs, based on the responses of 320 veterinarians surveyed between February and June 2022.

The majority of GPs worked only day shifts (207/236, 87.7%), where this shift pattern was the least common among EPs (10/83, 12.1%). There was greater variety in shift pattern among EPs (see Figure 2). Regarding weekend work, 37.1% (88/237) of GPs worked either “at least 1 day every weekend” or “1 in 2 weekends,” compared to 75.9% (63/83) of EPs (Figure 3). Most GPs (62.9%, 149/237) were not required to work on any public holidays, compared to 1.2% of EPs (1/83) (Figure 4). Most EPs were required to work more than 50% of all public holidays in their state/territory (56.7%, 47/83).

### Rosters

Among the respondents, 83.6% (195/236) of GPs worked a set and predictable roster pattern, while 61.5% (51/83) of EPs did not (*P* < 0.001). A small proportion of respondents indicated that they did not work a set and predictable roster pattern (92/319, 28.8%). Of these, 53 respondents (53/92, 57.6%) felt that they received their rosters well enough in advance to plan life outside of work, whilst 38 respondents (38/92, 41.3%) felt they did not. For veterinarians who felt that they received their rosters well enough in advance, the average notification period was 5.2 weeks (range 2 – 20 weeks), and for those who felt that they did not, the average notification period was 2.7 weeks (range 0–8 weeks), however some overlap existed between the two groups.

### Overtime and meal breaks

**Figure 5:**
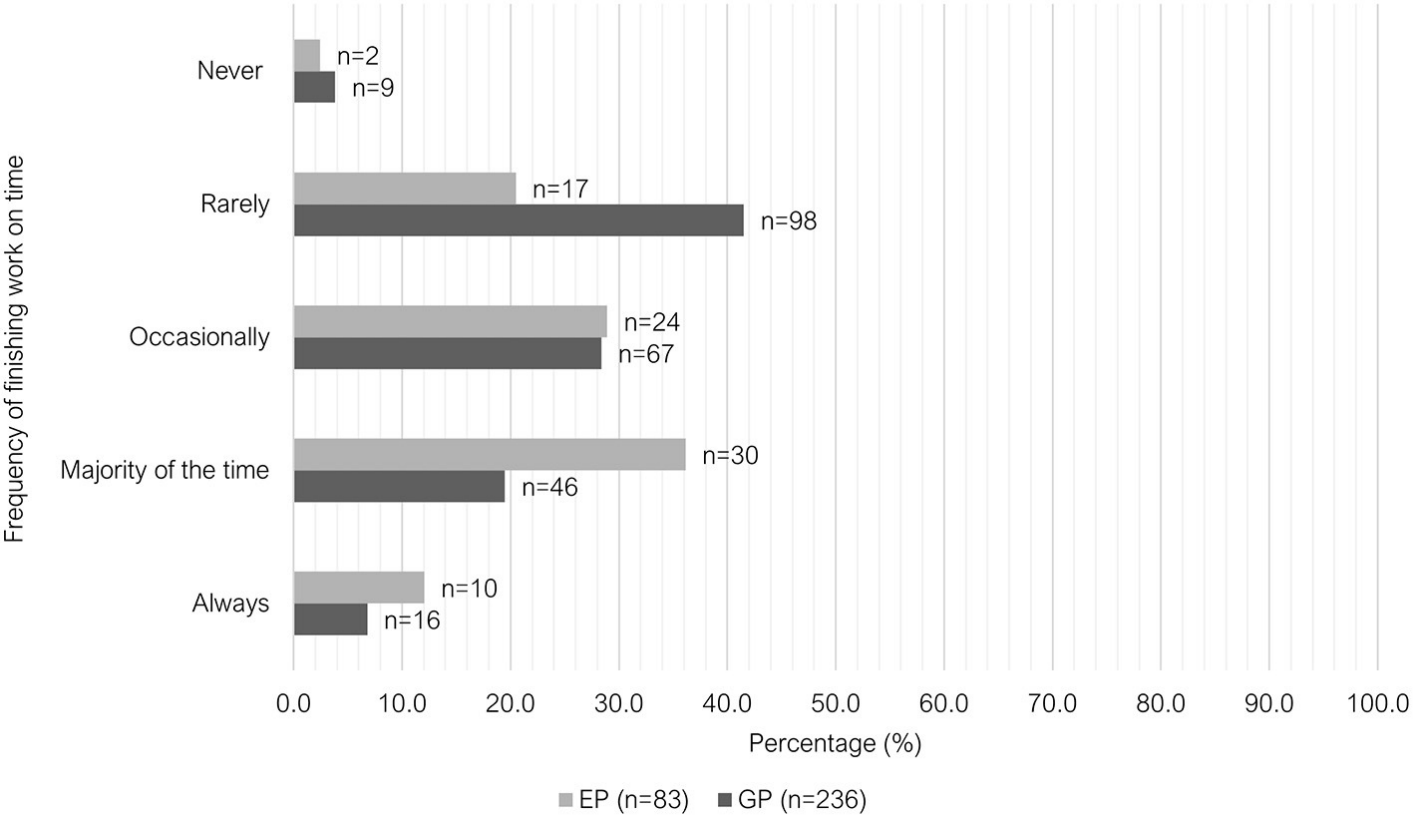
Clustered bar chart of the frequency of finishing on time for veterinary GPs and EPs, based on the responses of 320 veterinarians surveyed between February and June 2022.

**Figure 6:**
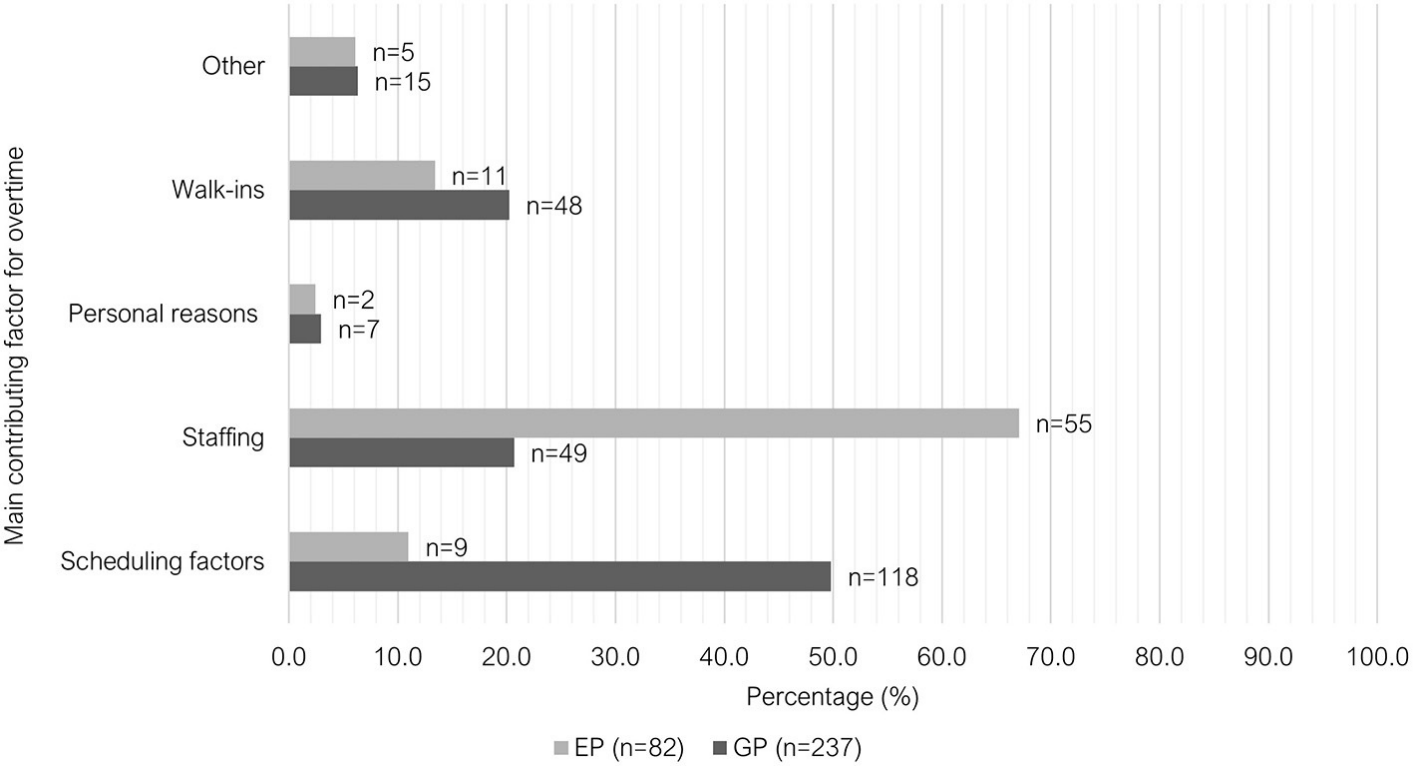
Clustered bar chart of the main contributing factor for overtime for veterinary GPs and EPs, based on the responses of 320 veterinarians surveyed between February and June 2022.

There were significant differences between the two groups (*P* < 0.001) in their ability to finish work on-time (Figure 5); main contributing factor to overtime (Figure 6) and meal break characteristics (Table 2). A large proportion of GPs indicated that they could finish work “rarely” on time (41.5%, 98/236); most EPs indicated that they could finish on time “majority of the time” (67.1%, 55/82).The main contributing factor to overtime for GPs was “scheduling factors,” with 49.8% (118/237) selecting this answer, whereas 67.1% (55/82) of EPs selected “inappropriate staffing or short staffing” as their main reason. Free-text responses were provided by respondents who selected “other” (*n* = 19). These responses were grouped under three categories: multifactorial (unable to select one main contributing factor); variable depending on the workday and additional, non-clinical responsibilities (Supplementary Table 3). The majority of EPs (69.9%, 58/83) indicated that they were “unable to take a meal break (< 30 min)” in the week leading up to survey. There was a lower proportion of GPs selecting this option (35.9%, 85/237); with the most selected option being “meal break 30 min−1 h, interrupted by work.”

**Table 2:** Frequency table comparing GPs and EPs (*P* < 0.001) for the question of “In the past week, the following statement best describes my meal breaks” (*n* = 320).

**Meal break characteristics**	**GP number**	**GP %**	**EP number**	**EP %**
Unable to take a meal break (< 30 min)	85	35.9	58	69.9
Meal break 30 min−1 h, interrupted by work	111	46.8	21	25.3
Meal break 30 min−1 h, uninterrupted	31	13.1	3	3.6
Meal break >1 h, interrupted by work	2	0.8	0	0.0
Meal break >1 h, uninterrupted	8	3.4	1	1.2

No significant difference (*P* = 0.230) was found between the two groups regarding the number of unpaid hours of work per work. On average 46.5% (148/318) of respondents performed <1 h of unpaid work per week, followed by 23.6% (75/318) of respondents who performed 1–3 h of unpaid work per week, then 15.1% (48/318) of respondents who performed 4–6 h of unpaid work per week. A relatively smaller number of respondents performed 7–10 h (17/318, 5.3%) or more than 10 h (30/318, 9.4%) of unpaid work per week.

### Staffing

No significant difference (*P* = 0.238) was found between GPs and EPs regarding the binary question “In the past week, I feel that my practice was appropriately staffed on most days of the week.” Most respondents indicated that their workplace was not appropriately staffed (62.5%, 200/320).

### Patient and client interactions

**Figure 7:**
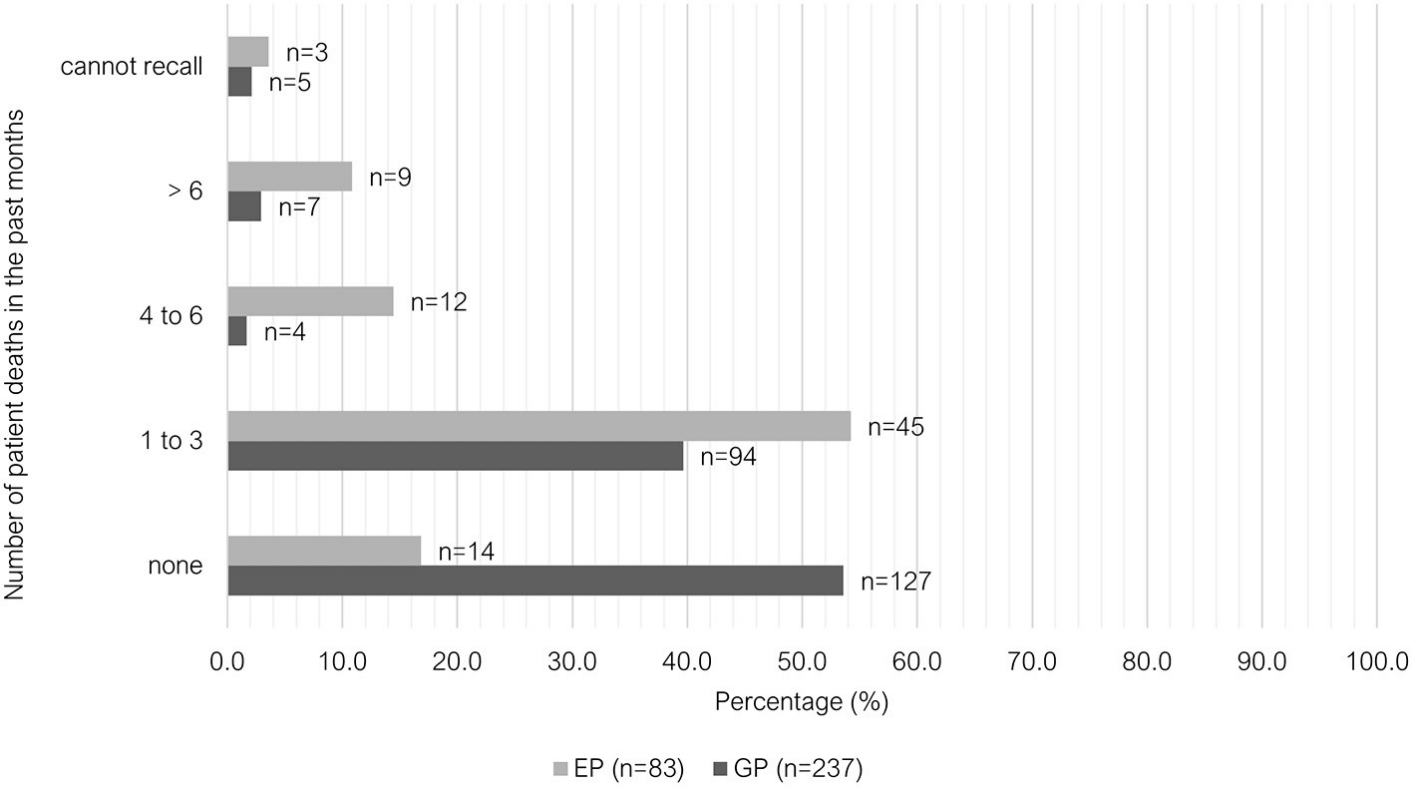
Clustered bar chart for the number of patient deaths encountered in the past months by veterinary GPs and EPs, based on the responses of 320 veterinarians surveyed between February and June 2022.

**Figure 8:**
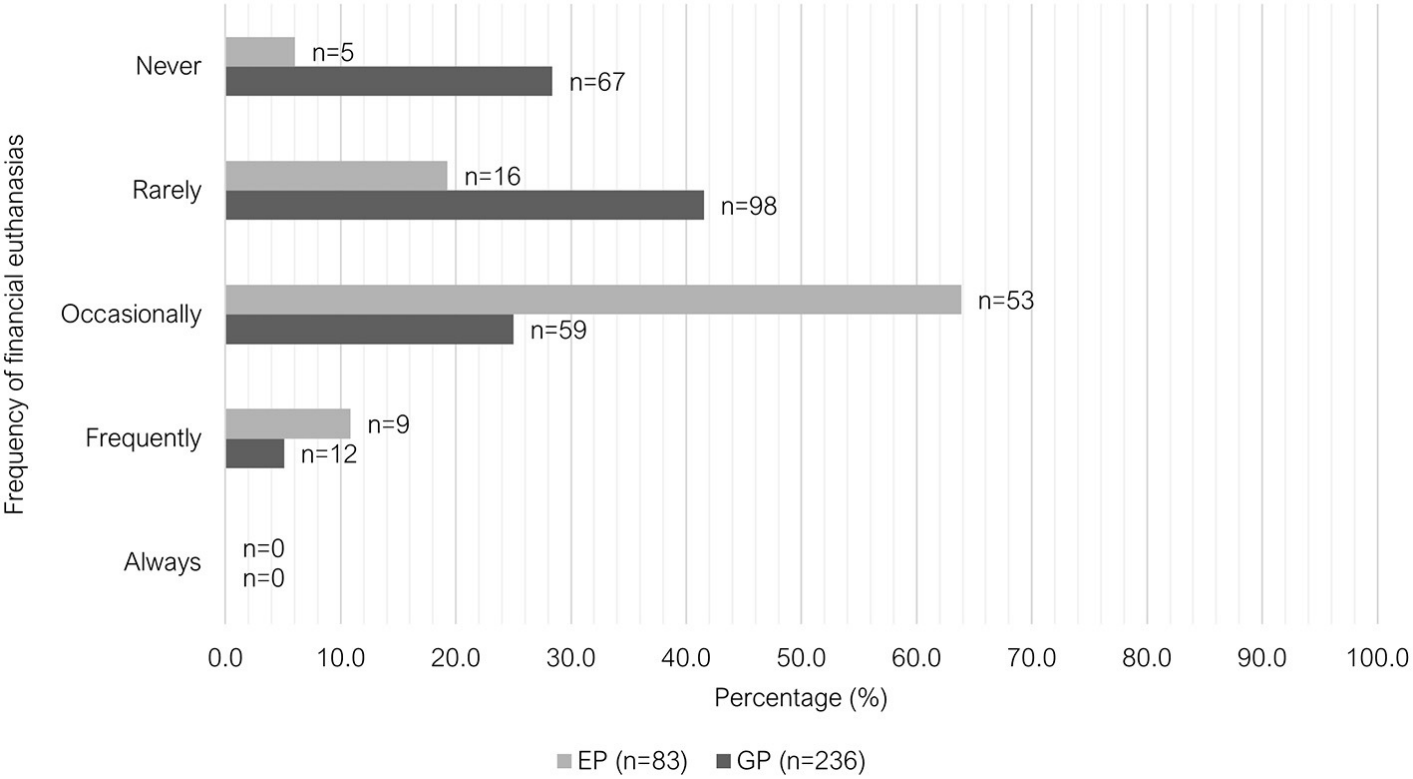
Clustered bar chart for the frequency of financial euthanasias performed by veterinary GPs and EPs, based on the responses of 320 veterinarians surveyed between February and June 2022.

Exposure to patient death including euthanasia was significantly different between GP and EP groups (*P* < 0.001). Most GPs did not experience patient death in the past month (53.6%, 127/237), whereas 54.2% (45/83) of EPs experienced 1–3 deaths (Figure 7). A similar proportion of GPs and EPs performed euthanasia 4 to 6 times in the past month (33.3% vs. 32.5%). However, EPs more frequently reported performing euthanasia six or more times in that previous month (50.6%, 42/83) compared to GPs (29.5%, 70/237). GPs reported “rarely” being requested to euthanize an animal where the primary reason was due to financial limitations (41.5%, 98/236), while EPs more commonly reported receiving these requests “occasionally” (63.9%, 53/83) (Figure 8).

**Figure 9:**
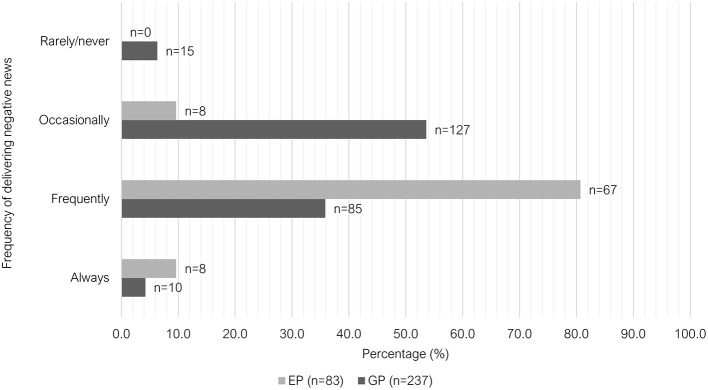
Clustered bar chart for the frequency of delivering negative news to clients by veterinary GPs and EPs, based on the responses of 320 veterinarians surveyed between February and June 2022.

**Figure 10:**
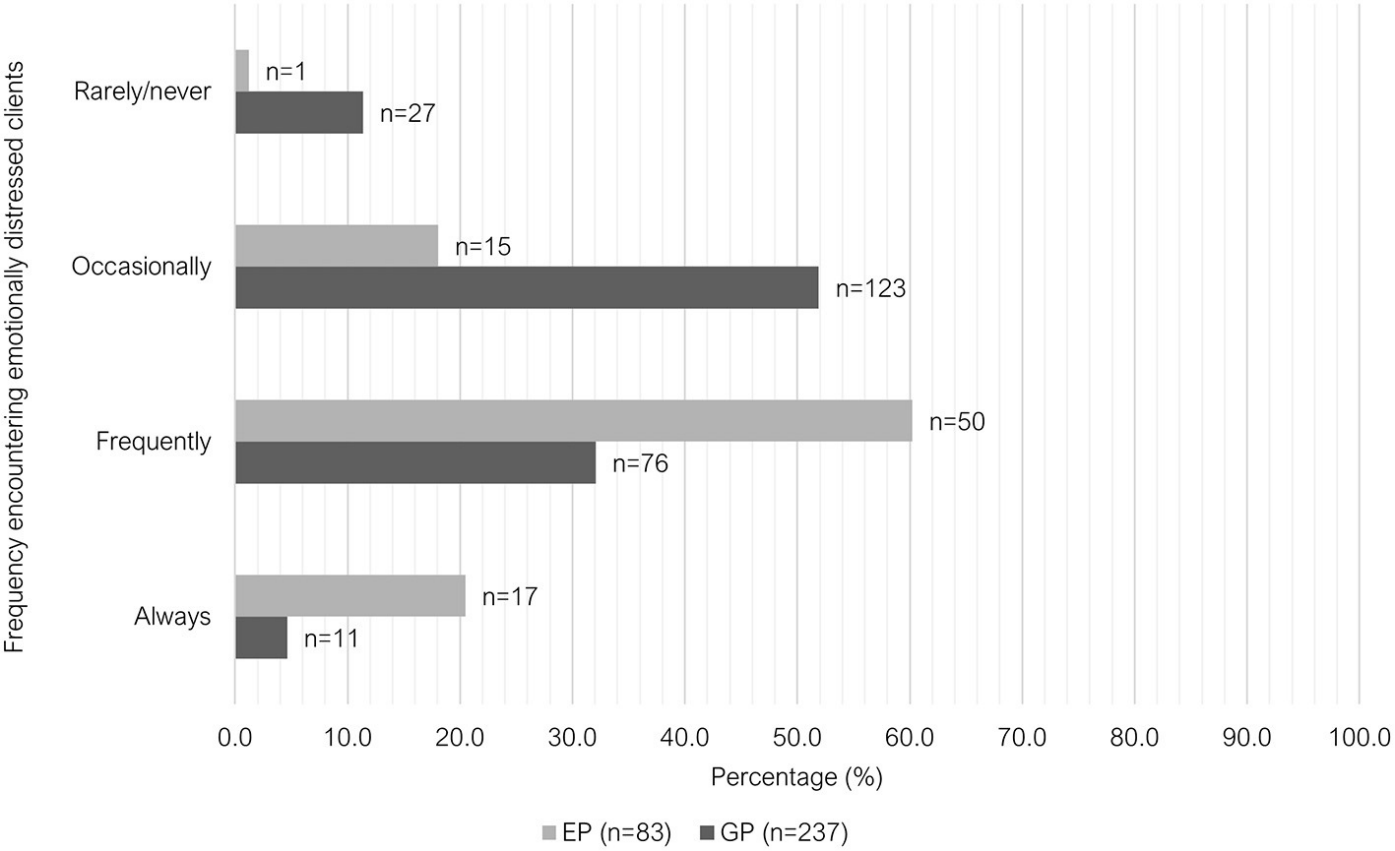
Clustered bar chart for the frequency of encountering emotionally distressed clients by veterinary GPs and EPs, based on the responses of 320 veterinarians surveyed between February and June 2022.

There were also significant differences between the two groups in the frequency they were required to deliver “negative news” (*P* < 0.001) and the frequency of interactions with “emotionally distressed clients” (*P* < 0.001). Just over one third (35.9%, 85/237) of GPs indicated that they were required to deliver “negative news” “frequently” to clients in the past week leading up to survey, whereas 80.7% of EPs selected this option (67/83). The most common response among GP vets for this question was “occasionally” (53.6%, 127/237) (Figure 9). A similar trend was observed when asked about the frequency of interactions with emotionally distressed clients (anxious, sad, or angry) within the past week; with most GPs indicating “occasionally” (51.9%, 123/237) and most EPs indicating “frequently” (60.2%, 50/83) (Figure 10).

**Figure 11:**
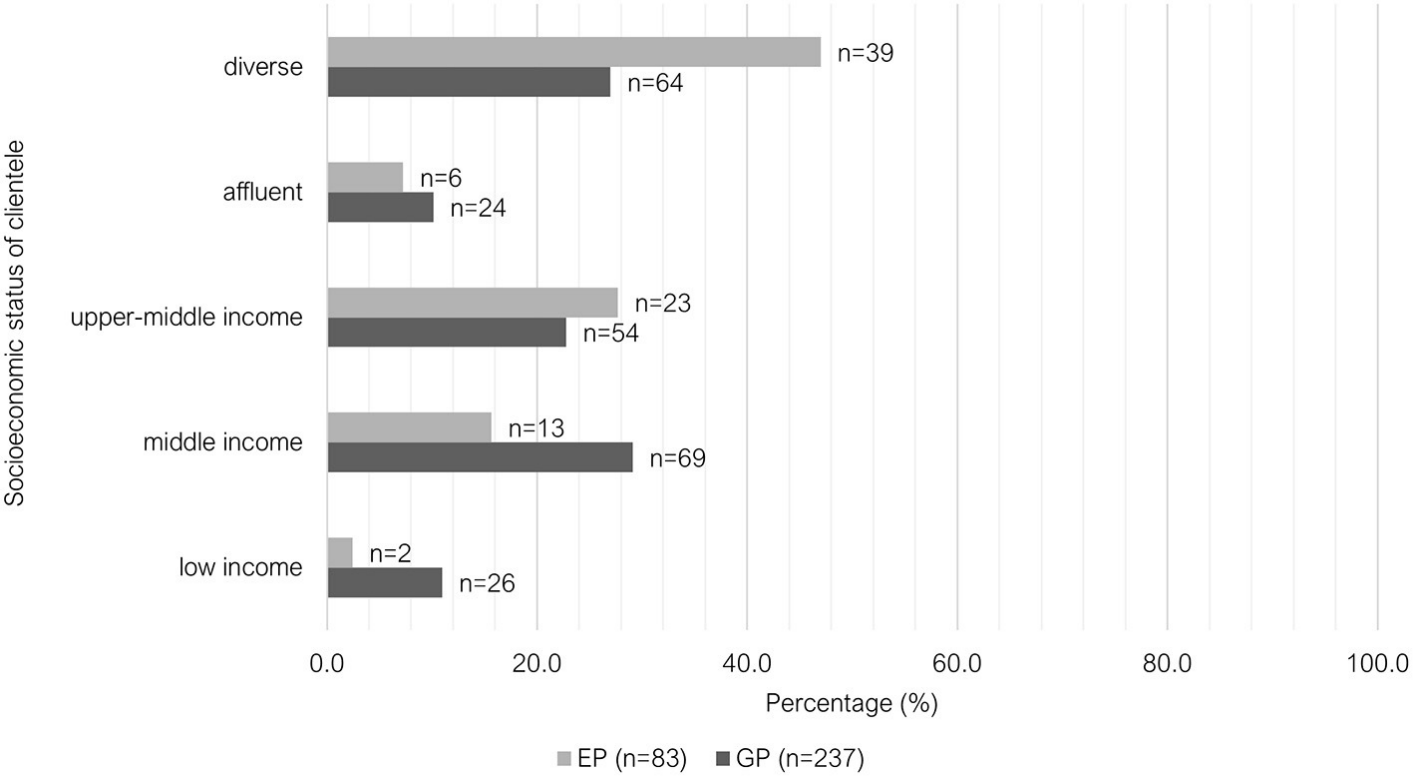
Clustered bar chart for the socioeconomic status of clientele of veterinary GPs and EPs, based on the responses of 320 veterinarians surveyed between February and June 2022.

The data was significantly different between the two groups for the reported perceived socioeconomic situation of their clientele (*P* < 0.001). The responses of GPs were more evenly distributed across middle income, upper-middle income and diverse, whereas most EPs indicated that their clientele come from a diverse socioeconomic background (47.0%, 39/83) (Figure 11). No significant difference (*P* = 0.087) was found between the two groups for client adherence (“receptive and compliant with your diagnostic and treatment recommendations”). Most respondents indicated that their clients were adherent to their recommendations for the “majority of the time” (84.1%, 269/320), followed by “occasionally” (12.5%, 40/320); “always” (2.5%, 8/320), and “rarely”/”never” (0.9%, 3/320).

### Workplace culture

No significant differences were found between GP and EP groups in regard to their workplace environment and experiences with workplace bullying. The majority of respondents indicated that both their colleagues and their management team were supportive and collegial (216/320, 67.5%). Just over one-fifth of respondents (20.6%, 66/320) indicated that their colleagues are supportive, but the management team is toxic; 5.6% (18/320) of respondents indicated the opposite (their colleagues are toxic, but the management team is supportive), while 6.3% (20/320) indicated that both their colleagues and the management team contribute to a toxic workplace environment. Furthermore, 52.8% (169/320) of respondents had not experienced or witnessed workplace bullying at their current workplace, while 19.1% (61/320) had experienced workplace bullying and 28.1% (90/320) had witnessed workplace bullying at their current workplace.

### Satisfaction and considerations for leaving

**Figure 12:**
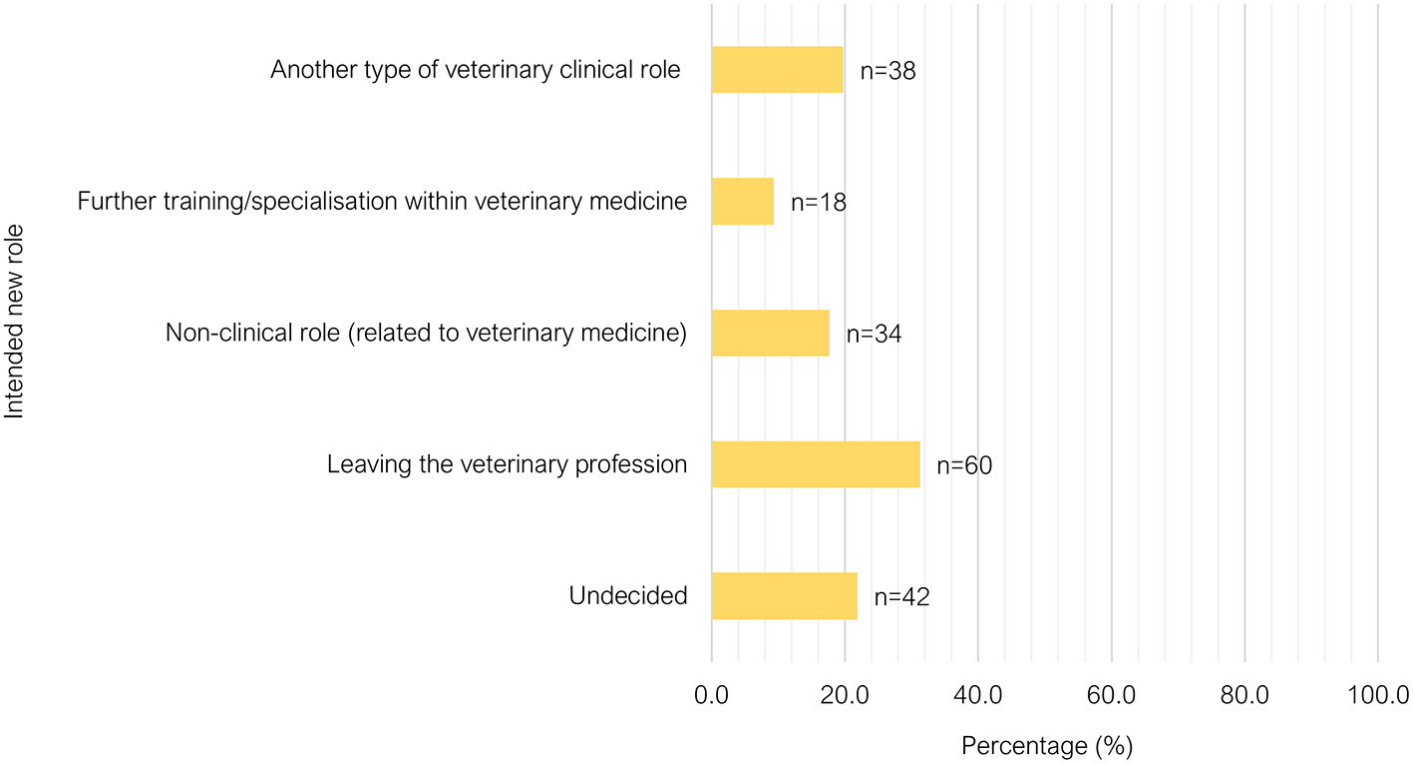
Bar chart of the new role considered by veterinarians who had considered leaving their current position in the past year, based on the responses of 320 veterinarians surveyed between February and June 2022.

Satisfaction in relation to remuneration (*P*=0.668) and workplace achievements (*P* =0.124), and considerations for leaving (*P*=0.567), were not significantly different between GP and ER groups. Three-quarters of respondents felt satisfied with what they had achieved at work in the past week (240/320). When asked about their satisfaction with their remuneration, 52.0% (166/319) of respondents were not satisfied. Most respondents had seriously considered leaving their principal area of practice within the past year (60.2%, 192/319). The respondents who had considered leaving were asked a follow-up question on what type of new role they would consider transitioning into (Figure 12), and the most favored response was “leaving the veterinary medicine profession” (60/192, 31.3%).

## Discussion

This is one of few survey studies to investigate the unique demographic and work-related factors of veterinary sub-groups. Significant differences were found between veterinary GPs and EPs in relation to demographics, shift pattern, hours of work, overtime, meal-breaks, and client-patient interactions. Some of these differences may predispose certain groups of veterinarians to negative mental and physical health outcomes. This information would be valuable for veterinarians considering transitioning into a small animal GP or EP role in metropolitan Australia. It would also benefit potential employers and managers, as addressing some of these modifiable factors could assist with staff satisfaction.

The age of GPs and EPs were significantly different in this study. The age structure of GPs, matched more closely with the reported age breakdown from the 2021 Australian Veterinary Association (AVA) Workforce survey (16.6% under 30 years old, 27.7% 30–40 years old, 21.0% 40–50 years old, 34.7% 50 years and older) (38). In general, EPs were younger, with less years of experience in their field. In previous studies, increasing age or years of experience has been associated with protection against stress and burnout (8, 25, 39). These trends raise concerns about the longevity of careers in emergency practice and subsequent effects on staff turnover. A variety of factors could contribute to this including the propensity for shift work, weekend/public holiday work, irregular hours, lack of predictability (34), lack of ability to take meal breaks and more frequent negative client and patient interactions.

Previous research has identified female veterinarians as more vulnerable to stress, anxiety and burnout compared with the general population due to tendencies toward negative coping strategies such as rumination or escapism (40). But, female veterinarians reported no significant difference in work satisfaction when compared to male veterinarians (41). Buchanan et al. (42) suggested that gender may not be the main determinant of vulnerability to negative mental health outcomes; instead, a combination of gender and parenthood is important. Indeed, female veterinarians with two or more children were less likely to suffer from anxiety and depression, compared with women who did not have children. Another study showed that in the veterinary profession, mothers were more satisfied with work than fathers, however childless men were more satisfied than childless women (41). This suggests that the influence of gender on mental health is nuanced.

One of the main differences between general and emergency practices is the requirement in operating hours. Emergency centers typically provide patient care 24 h a day, 7 days a week. Unsurprisingly this is reflected in the shift pattern found in this study. EPs indicated that they worked a greater variety of shift patterns, with more night shifts compared to GPs. Previous literature has shown shift work, especially night-time work that contradicted the natural circadian rhythm, was associated with poorer mental health and physical health outcomes (43–45). Another concern for shift workers is social isolation due to conflicting schedules with family and friends. A 2014 study in healthcare workers found that burnout was found to be significantly more frequent in shift workers compared to non-shift workers (46). This study found that when shift workers were satisfied with their schedule and experiencing lower impacts on private life, then they had a lower level of burnout and higher level of satisfaction compared to their counterparts (46). In our survey group, EPs performed more weekend and public holiday work compared to GPs. An Australian study found that weekend work was associated with less shared leisure time in all family types investigated (47). Another study showed lower family satisfaction from workers performing non-standard hours, especially evening and night shifts (48). As personal circumstances vary between individuals, it could be beneficial to employee satisfaction for employers to offer more choices when it comes to weekend and public holiday work (for example, asking employees to choose one to two public holidays a year to take off where public holiday work is required).

Shift work, weekend and public holiday work are unavoidable for emergency practices; therefore, employers should focus on rostering strategies that minimize health and safety risks. Shift duration of 12 h or longer was associated with reduced alertness, increased medical errors and increased risk of burnout and occupational injuries (31, 49, 50). In these studies, the factor most consistently associated with increased injuries was quick return, mostly defined as an interval between shifts of <11 h in duration (49). The negative effects of quick return were particularly pronounced following a night shift. Working for >3 consecutive night shifts has also been associated with an increase in workplace injuries (51, 52). Injuries were more likely to occur on the first night shift of a rotating roster, as fatigue and drowsiness were more pronounced (the “first night effect”) (51, 52). Some have argued that a fixed night-shift pattern would be able to minimize the first night effect as well as to allow for re-entrainment of the circadian rhythm (53). However, re-entrainment only occurred in a small proportion of shift workers, with most still suffering from reduced quality of sleep (54). Whether a fixed night-shift pattern results in more satisfaction among employees also remain controversial (55–57). As variabilities in personal circadian rhythms and sleep patterns exist, satisfaction among employees may improve if given the freedom of choice (45). “Forward-rotating” rosters, whereby a day shift was followed by an evening and then a night shift, have been reported to be better for sleep than “backward-rotating” rosters, where a night shift was followed by an evening shift, then a day shift. The forward-rotating pattern was associated with improved duration and quality of sleep (49, 58). The forward-rotating pattern includes longer intervals between shifts when compared to backward-rotating systems.

In our study group, not all employers followed the Australian Animal Care and Veterinary Services Award 2020 requirement for roster notification periods. Under section 13.3b, the Award stipulates that “daily work rosters will be published at least 1 month in advance” for veterinary surgeons (35). A large proportion of respondents did not feel that their roster notification period was adequate (41.3% of respondents). The average notification period was 2.7 weeks in the group that were dissatisfied with their roster notification period, which is well below the minimum requirement. This highlights the critical need for Australian veterinary employers to extend the roster notification period to allow personal planning and a sense of predictability. It is possible that employers have struggled to fill rosters in advance due to the current shortage of veterinarians (59). However, not being advised of one's roster in advance may be a source of dissatisfaction among veterinarians and has the potential to exacerbate this shortage. Based on the findings in this survey, we recommend to employers to notify employees of their roster as far ahead in advance as possible, ideally 5 weeks or more.

We found GPs were more prone to overtime compared to EPs. This difference could be due to the continuous nature of emergency practices, where consults that occur close to finish time can be seen by the next available veterinarian, allowing the individual to allocate more time to finish paperwork and following up on pending diagnostics. This would not be possible in a general practice with a set closing time, and where intentional scheduling efforts are required. This is reflected in the finding that most GP respondents' main contributing factor to overtime was “scheduling” and evidenced further by free-text comments. We recommend that employers review scheduling and ensure that time is scheduled or protected to complete work-related tasks including review and treatment of hospitalized patients, following up and reporting of results, owner communication and medical record writing. For general practices wishing to accommodate walk-ins, there is additional complexity for appropriate scheduling strategies. In human healthcare, mathematical models have been used to develop appointment scheduling systems to accommodate walk-in appointments and balance clients who do not attend appointments (60–62). It may be useful to pilot such systems in veterinary clinical contexts.

EP respondents indicated that low staffing levels were the key contributing reason for overtime. A solution for inappropriate staffing will be difficult given the current Australian veterinary workforce shortage. The 2021 AVA Workforce Survey showed 77.5% of respondents working in practice were aware that their practice was advertising for a veterinarian vacancy. Concerningly, 30.6% of vacancies took more than 12 months to fill or were still not filled at the time of survey (26). This problem has been compounded in recent years due to the estimated 10.0% increase in Australian pet ownership during the COVID-19 pandemic (63, 64). This issue has now been raised to a government level, and at the time of writing there is an ongoing parliamentary enquiry into the veterinary workforce shortage in the state of New South Wales (59).

Revised scheduling may partially alleviate the pressure in general practices. However, the expectation for emergency practices to accommodate all emergencies presented is a more challenging scenario in the face of workforce shortages. In addition to increasing staff overtime hours, understaffing lead to increased wait times and compromised patient care (65). In many cases, emergency hospitals have had to divert patients to alternative facilities (including general practices) (66–69). The authors of the current study have directly experienced varying diversion or at-capacity strategies across different emergency hospitals. Concerns have been raised by both staff and management over the ethics and legality of diverting care or refusing care to emergent patients. Lack of coordination of care diversion can lead to multiple emergency practices diverting patients simultaneously, leaving a whole geographical region without access to emergency veterinary care. A 2022 US study investigating the impact of the COVID pandemic on companion animal care recommended increased collaboration and communication across veterinary clinics to increase access to veterinary care, and investigating the use of telemedicine to relieve workload (70). Veterinary boards can publish more specific guidelines regarding care diversion to clarify the responsibilities of veterinary services and individual veterinarians where surge capacity is exceeded.

The majority of EPs were unable to take a meal break, while most GPs were able to take a 30 min to 1 h break, though this was frequently interrupted. Skipped or interrupted breaks lead to higher levels of stress, burnout and exhaustion (emotional and physical) (71–73). It can also impair work performance and induce errors by instigating provider fatigue (74). As mentioned previously, general practices should focus on optimizing scheduling to ensure that staff are able to take appropriate meal breaks. The nature of emergency practice may necessitate veterinarians prioritizing patient care over meal breaks. However, rostering to allow staggered or overlapping shifts of emergency consulting veterinarians can allow staff members to take appropriate breaks in emergency settings (75).

Socioeconomic status (SES) of clients may be linked to financial constraints, and financial constraints imposed by clients may limit the type of veterinary care that veterinarians can provide. Such limitations are a source of self-reported burnout and moral stress among veterinarians (4, 76). Our results suggest that GPs work with a more homogenous SES group, where EPs serve clients from a more diverse socioeconomic background. This could mean that EPs are required to vary their approaches more frequently to devise a diagnostic and treatment plan that caters for the diversity of socioeconomic backgrounds they encounter, leading to increased cognitive load (31). This study showed that EPs were more likely to interact with emotionally distressed clients, deliver negative news, be exposed to more patient death and perform more euthanasia, including economic euthanasia. The act of euthanasia itself can be associated with grief, but not necessarily associated with negative mental health outcomes (2, 77, 78). On the contrary, being able to provide a “good death” can increase a sense of wellbeing and job satisfaction (79). However, euthanasia decision-making was a greater source of stress (79). Focusing on increased training in navigating end-of-life decision-making can be a focus for veterinarians in training, whilst employers can focus on offering additional de-briefing support and ongoing communication training. Increased exposure to financial euthanasia can lead to moral distress (4, 80, 81). For the purposes of this discussion, we have used the term “financial euthanasia,” however, some authors may argue that a more appropriate term would be “humane killing” (82). During the COVID-19 pandemic, many clinics implemented protocols to reduce client contact resulting in non-contact euthanasia, further exposing veterinary team members to greater ethically challenging situations (83).

In the literature, emergency physicians are reported to suffer from increased vicarious trauma and secondary traumatic stress due to routine encounters with stressful clinical situations such as the deaths of patients and delivering bad news (84–86). The current survey show that EPs are exposed to similar workplace stressors, therefore may be likely to suffer from similar psychological impacts. However, the prevalence of vicarious trauma, post-traumatic stress disorder and secondary traumatic stress has not been evaluated in this group to date. Further research into the trauma experienced by emergency veterinarians may assist in developing mitigation strategies.

In this current study, more respondents were dissatisfied with their remuneration compared to the 2021 AVA Workforce Survey. This could reflect the specific population of veterinarians enrolled in this survey, small animal GPs and EPs working in metropolitan regions compared to the broader veterinary population surveyed by the AVA. More specific remuneration data regarding this group is needed to determine if improved remuneration could assist with mitigating burnout.

In light of the current workforce shortage of veterinarians, it is concerning that almost two thirds of respondents had considered leaving their principal area of practice within the past year, with a third wishing to leave the veterinary profession altogether. This finding was in line with a previous 2017 study that showed 27% of sampled experienced veterinarians were at risk of leaving the industry in the next 3 years (87) A study performed by Montoya et al. (20) interviewed 26 former veterinarians on their reasons for leaving clinical practice. This qualitative study showed that there were a variety of interplaying personal reasons (negative personal thoughts, physical and mental health) and work-related factors (employment conditions including relationship with colleagues, remuneration, working hours and negative clinical outcomes) (20). Another possible reason for this high prevalence could be due to non-response bias, given this survey was completely voluntary with no incentives provided. Future research could investigate the impacts of modifying exposures to workplace factors.

## Limitations

One of the limitations to this study is that a response rate could not be calculated as it was impossible to quantify the population of people who saw the survey but did not click on the survey link. Another key limitation is the voluntary nature. This survey relies on self-selection, meaning respondents chose whether or not to participate. This can introduce non-response bias as individuals who feel strongly about a particular topic are more likely to respond, while those who are indifferent or have opposing views may opt out. As a result, our survey results may not accurately reflect the broader population. Additionally, it is possible that some terms or phrases in the survey, such as “toxic workplace” or “collegial/supportive workplace” could have been interpreted differently by respondents or may have been leading. We piloted the survey with a diverse cohort to minimize leading questions, but acknowledge that the phrasing could be a source of bias in our results.

Strict anonymity was one of the goals of this study design to protect the privacy of respondents and to minimize social desirability bias. One disadvantage of anonymous surveys is the inability to clarify responses. Lastly, a very specific group was targeted for this survey study – small animal GPs and EPs in metropolitan Australia. Hence, our findings may not apply to other groups of veterinarians within Australia and internationally. It would be an interesting area of future research to assess if these differences we found between veterinary GPs and EPs in Australia also apply internationally.

In conclusion, many significant differences were found between veterinary GPs and EPs practicing in metropolitan regions of Australia. We found that GPs tended to be older, with more years of experience in their field when compared to the EP group. The EP group performed more hours of work each week but worked less overtime. GPs indicated that the main contributing factor to overtime was due to scheduling factors, whereas EPs indicated staffing issues as the main reason. EPs were not able to take meal-breaks, while GPs' meal-breaks were commonly interrupted by work. EPs were more likely to work variable shift patterns and perform weekend or public holiday work compared to GPs. EPs were more frequently exposed to patient death, euthanasia (including for financial reasons), emotionally distressed clients and delivering negative news. Both groups indicated that most work environments were collegiate and supportive, and a minority reported toxic colleagues (11.8%) or management teams (26.9%). Just under one-half of respondents reported having witnessed or experienced workplace bullying. Of our respondent group, 52.0% were not satisfied with their remuneration. Some of these could represent distinct risk-factors contributing to burnout among these subgroups, a topic explored in our subsequent paper. Desires to leave their role was prevalent among this survey group.

## Data availability statement

The datasets presented in this article are not readily available due to Conditions of The University of Sydney Human Research Ethics Committee. Requests to access the datasets should be directed to anne.quain@sydney.edu.au.

## Ethics statement

The studies involving humans were approved by the University of Sydney Human Research Ethics Committee. The studies were conducted in accordance with the local legislation and institutional requirements. The participants provided their written informed consent to participate in this study.

## Author contributions

KL: Conceptualization, Formal analysis, Investigation, Methodology, Writing – original draft, Writing – review & editing. EM: Conceptualization, Methodology, Supervision, Writing – review & editing. MM: Investigation, Supervision, Writing – review & editing. EH: Data curation, Methodology, Writing – original draft. AQ: Conceptualization, Methodology, Supervision, Writing – review & editing.
