# Supplementary material for: A comparison between veterinary small animal general practitioners and emergency practitioners in Australia. Part 1: demographic and work-related factors
Source: Front Vet Sci. 2024 Mar 21;11:1355505. doi: 10.3389/fvets.2024.1355505 (PMC10993158; doi:10.3389/fvets.2024.1355505)
Supplement: Supplementary file 1 [file Table_1.pdf]

frontiers

Supplementary Material

Supplementary Table 1. Survey Questions - A survey study of burnout and risk factors in Australian emergency and general practice veterinarians.

Questions	Options
I have read the participant information sheet. I am over the age of 18 and consent to participate in this study.	Yes<br>No
I am a small animal veterinarian, who is currently practicing in a metropolitan area within Australia. I am currently working in either emergency or general practice.<br>Definition of metropolitan area according to the Rural, Remote and Metropolitan Areas Classification (RRMA) 1991: an urban centre with a population of 100 000 or more. If you are unsure whether you are working in a metropolitan area, you can check your RRMA classification by searching your suburb on: https://www.health.gov.au/resources/apps-and-tools/health-workforce-locator/health-workforce-locator	Yes<br>No
**Part 1/3 – Work Related Questions**	
What is your principal area of practice?<br>*Place of work where you have spent more than 60% of your time in the last 3 months.	Small animal general practice<br>Small animal emergency practice
How long have you worked in your principal area of practice (GP / emergency)?<br>*In years (e.g., 6 months = 0.5 years)	(Free text response)
What is your position in your practice?	Associate veterinarian<br>Management<br>Practice owner
On average, how many hours do you work per week?<br>*Include all paid and unpaid overtime	Less than 35 hours per week<br>35 - 40 hours per week<br>40 - 50 hours per week<br>50 - 60 hours per week<br>Greater than 60 hours per week
Which of the following best describe your shift pattern in the past month?	Only day shifts (work hours largely fall between 7am - 7pm)<br>Only night shifts (work hours largely fall between 7pm - 7am)<br>Majority day shifts (more than 70%)<br>Majority night shifts (more than 70%)<br>None of the above (less than 70% of either day or night shift)
How often do you work on the weekend?	At least one day every weekend<br>1 in 2 weekends<br>1 in 3 weekends

	1 in 4 weekends<br>Infrequently (less than 1 in 4 weekends)<br>I never work on the weekend
Which of the following statements best describe your work schedule on a public holiday?<br>*Please answer with regard to the public holidays observed in the state/territory in which you work	I am not required to work on a public holiday.<br>I am able to choose if I want to work on a public holiday.<br>I am required to work less than 50% of public holidays.<br>I am required to work 50% or more public holidays.<br>I am required to work every public holiday
My roster pattern is set and predictable.	Yes<br>No
I receive my roster well enough in advance to be able to plan my life outside of work.	Yes<br>No
In relation to the previous question, on average how many weeks in advance do you receive your roster?<br>(In weeks)	(Free text response)
On average, how many unpaid hours do you work per week?	Less than 1 hour<br>1 - 3 hours<br>4 - 6 hours<br>7 - 10 hours<br>Greater than 10 hours
How frequently are you able to finish all required work within your rostered shift time?	Always<br>Majority of the time.<br>Occasionally<br>Rarely<br>Never
What is the main contributing factor to not finishing on time?	Scheduling factors<br>Inappropriate staffing or short staffing<br>Personal reasons<br>Equipment/software inadequacies<br>Pressure to fit in walk-in consults<br>Other
If other, please specify.	(Free text response)
In the past week, the following statement best describes my meal breaks.<br>*Please answer based on what happened for the majority of the week.	Unable to take a meal break (< 30 minutes)<br>Meal break 30min - 1 hour, interrupted by work<br>Meal break 30min - 1 hour, uninterrupted<br>Meal break greater than 1 hour, interrupted by work<br>Meal break greater than 1 hour, uninterrupted

In regards to your current place of work, how would you describe the socioeconomic situation of the majority of your clientele?	Low income and pensioners<br>Middle income<br>Upper-middle income<br>Affluent income<br>Diverse (even mix of all of the above)
Think back to the past week, how often are your clients receptive and compliant with your diagnostic and treatment recommendations?	Always<br>Majority of the time<br>Occasionally<br>Rarely<br>Never
How many times did you experience patient death (non-euthanasia) in the past month?	None<br>1-3 times<br>4-6 times<br>Greater than 6 times<br>Do not recall
How many euthanasias were you requested to perform in the past month?	None<br>1-3<br>4-6<br>7-10<br>Greater than 10
In relation to the previous question, how many euthanasias were you requested to perform where the primary reason was due to financial limitations?	Always<br>Frequently<br>Occasionally<br>Rarely<br>Never
In the past week, how often were you required to deliver negative news* to clients regarding their pet's health?<br>*negative news can be serious illness, life-threatening conditions, terminal illness or recommendation for euthanasia.	Always<br>Frequently<br>Occasionally<br>Rarely<br>Never
In the past week, I feel that my practice was appropriately staffed on most days of the week.	Yes<br>No
Which of the following statements best describe your current workplace environment?	Both my colleagues and the management team are supportive and collegial.<br>My colleagues are supportive and collegial, but I am pressured by a toxic management team.<br>My colleagues are toxic, but my management team is supportive.<br>Both my colleagues and the management team contributes to a toxic workplace environment.
In your current place of work, have you experienced or witnessed workplace bullying?	Experienced workplace bullying<br>Witnessed workplace bullying<br>No, did not experience or witness workplace bullying

In the past week, I feel satisfied with what I have achieved at work.	Yes<br>No
In the past week, how often were you interacting with emotionally distressed clients?<br>*For example: anxious, sad or angry clients.	Always<br>Frequently<br>Occasionally<br>Rarely<br>Never
I am satisfied with my renumeration for the work that is required of me.	Yes<br>No
In the past year, have you seriously considered leaving your principal area of practice (GP/Emergency)?	Yes<br>No
After leaving your current role, what type of work will you consider transitioning into?	Another type of clinical role in veterinary medicine<br>Pursue further training or specialisation within veterinary medicine<br>Non-clinical role, but related to veterinary medicine<br>Leaving the veterinary medicine profession<br>Undecided
**Part 2/3 – Burnout Measurement**<br>The following three sets of similar questions are in relation to how you feel about your work, client interactions and personal life.	
**Work burnout**	
Do you feel worn out at the end of the working day?<br>Are you exhausted in the morning at the thought of another day at work?<br>Do you feel that every working hour is tiring for you?<br>Do you have enough energy for family and friends during leisure time?<br>Is your work emotionally exhausting?<br>Does your work frustrate you?<br>Do you feel burnt out because of your work?	Never<br>Seldom<br>Sometimes<br>Often<br>Always
**Client burnout**	
Do you find it hard to work with clients?<br>Does it drain your energy to work with clients?<br>Do you find it frustrating to work with clients?<br>Do you feel that you give more than you get back when you work with clients?<br>Are you tired of working with clients?<br>Do you sometimes wonder how long you will be able to continue working with clients?	Never<br>Seldom<br>Sometimes<br>Often<br>Always
**Personal burnout**	
How often do you feel tired?<br>How often are you physically exhausted?<br>How often are you emotionally exhausted?	Never<br>Seldom<br>Sometimes

How often do you think: “I can’t take it anymore”?<br>How often do you feel worn out?<br>How often do you feel weak and susceptible to illness?	Often<br>Always
**Part 3/3 – Demographic characteristics**	
Which gender do you identify with the most?	Female<br>Male<br>Other
What is your age?<br>(In years)	(Free text response)
What is your household make up?	Single, no dependent children<br>Single, with dependent children<br>Married / de-facto relationship, no dependent children<br>Married / de-facto relationship, with dependent children
Thank you for answering the survey questions. If you feel that there are reasons for burnout that is not captured in this survey or if you have any general feedback, please comment in the box below:	(Free text response)
If you are interested in receiving a copy of the final results from this study, please email: kun.li@sydney.edu.au<br>If any of the questions in this survey has evoked feelings of distress, we would recommend seeking assistance from professional organisations or a health professional, including:<br>• Beyond Blue Support Services - Ph: 1300 22 4636<br>• Lifeline Australian - Ph: 13 11 14<br>• Your doctor<br>I understand that by submitting my responses I am consenting for the information I have entered to be used for research purposes.	

Supplementary Table 2. Organisations that agreed to distribute the link to an online survey of burnout and risk factors in Australian emergency and general practice veterinarians.

**Organisation Type**	**Name of Organisation**
State or territory veterinary practitioners board	Veterinary Practitioners Board of New South Wales<br>Australian Capital Territory (ACT) Veterinary Practitioners Board<br>Veterinary Practice Board of Western Australia<br>Veterinary Practitioners Board of Victoria
Veterinary peak body	Australian Veterinary Association
College	Australian and New Zealand College of Veterinary Scientists (ANZCVS)<br>ANZCVS – Emergency and Critical Care Chapter
Employers	Greencross Vets<br>VetPartners
Continuing education provider	Centre for Veterinary Education
Social media group	Vets Stay Go Diversify – Down Under

Supplementary Table 3. Free-text responses from respondents who selected "other" for the question – "What is the main contributing factor to not finishing on time?" (n=19).

**Common themes**	**Original free-text responses**
Multifactorial	"All of the above"<br>"Combination of walk-ins, tight consult times, computer problems"<br>"Its little bits of everything - some clients late, some clients being squeezed in, some software being slow/not working, never quite enough staff. All the small things add up to frequently not finishing on time"<br>"Many reasons. It is not a profession where we should expect to finish on time every shift - that will only lead to unfulfilled expectation and disappointment"<br>"Animals needing treatments, owners called, etc"<br>"Walk in consults, sometimes short staffed,"
Variable depending on the workday	"Scheduling, walk-ins, internet issues to write up notes, inadequate staffing all contribute but the proportion that they contribute varies with the day"<br>"A combination of those factors; depending on the day."<br>"Busy and unpredictable caseload"<br>"Nature of emergency work"<br>"Unexpected, involved cases"<br>"Unexpectedly complicated cases"
Additional non-clinical responsibilities	"Trying to fit in management work on top of consults etc"<br>"Too many responsibilities as veterinary director - doing my work, looking after other people, problem solving and management duties"<br>"The boss, trying to provide support and filling in gaps when short-staffed"<br>"Management tasks"<br>"Management issues taking up clinical time"<br>"Helping other vets"<br>"Owner, always admin and other stuff have to do"
